# Self-Destroyers

**Published:** 1858-10

**Authors:** 


### SELF-DESTROYERS.

WE do not hold the drunkard guiltless, who, by his infirmity, has disabled himself, disgraced his friends, and beggared his family. The convict who cut off his right hand, in order to avoid work, is not excused from labor, but receives our reprobation: and if, by any form of inconsideration or recklessness, we bring on ourselves an incapacity for performing the duties of life which devolve upon us, we are responsible for their discharge towards the party to whom those duties belong.

A clergyman of talent and culture, in the very prime of life, according to that excellent paper, *The Boston Recorder*, advertises that, being by ill-health incapable of performing ministerial duty, he is willing to do almost any thing for a moderate compensation; he is ready to preach an occasional sermon, prepare an essay, write an editorial, copy papers, read proof, prepare manuscripts for the press, keep books, deliver lectures, teach school, give lessons in elocution, reading or speaking—in any of which ways he hopes he might give satisfaction, and would do his best to please. We have no reason to question this gentleman's ability for any of these offices, and do certainly regret, when, in our own country, there are thousands of persons, singly and whole communities, who do not hear a gospel sermon in a year, that one so competent should be disabled. But, to use a common phrase, he has no business to be sick. In other words, his being a sick man is not a necessity, most likely. People do not get sick without a cause, except in rare cases; and that cause is, very generally, within themselves, resulting from inattention, ignorance, or recklessness, either on the part of parents, teachers, or themselves. It is a very poor excuse for a man to say that he cannot pay a debt—the declaration becomes insulting to the creditor, when that inability is the result of improvidence, or actual extravagance. When any man is disabled, by sickness, from discharging his duty to himself, his family, or society, the question should at once be, Is it from Heaven, or of men? Not of the former; for it is said, He does not willingly afflict the children of men: consequently, sickness is not of His sending. It is the result of causes within ourselves. In a literal sense, as well as a moral, it is true: "O Israel! thou hast destroyed thyself!" In plainer terms, disease is not sent upon us; we bring it on ourselves—and health is a duty.

